# Grand Challenges in Global Health: Ethical, Social, and Cultural Issues Based on Key Informant Perspectives

**DOI:** 10.1371/journal.pmed.0040268

**Published:** 2007-09-11

**Authors:** Kathryn Berndtson, Tina Daid, C. Shawn Tracy, Anant Bhan, Emma R. M Cohen, Ross E. G Upshur, Jerome A Singh, Abdallah S Daar, James V Lavery, Peter A Singer

## Abstract

The authors interviewed key informants from the developing world and the Grand Challenges investigators to explore their ethical, social, and cultural concerns about the program.

In 2005, we launched the Ethical, Social and Cultural (ESC) Program for the Grand Challenges in Global Health (GCGH) initiative, as described in the first article in this series [1]. To identify key ESC issues, we conducted group discussions with investigators and program officers from the GCGH projects, as well as interviews with developing world experts. Our approach is shown in Box 1.

**Box 1. Our Approach**

We identified ESC issues in the GCGH initiative through a sequential process of document analysis, group discussions with investigators and program staff, and interviews with experts from the developing world. We did not define ESC issues for our respondents. Rather, we included in our analysis any issue that a respondent characterized as ethical, social, or cultural. This method produced a broad and inclusive set of ESC issues appropriate for a large-scale project with diverse needs.

In April and May 2005, we reviewed the proposals for the GCGH projects to identify ESC issues. Some of the proposals explicitly identified ESC issues and provided specific strategies to address them. In our preliminary review, we identified implicit ESC issues in the proposals. Each proposal was reviewed in depth by a member of our team, and we developed a common framework for ESC issues across the proposals.

In November 2005, at the GCGH kick off meeting in Seattle, Washington, we held group discussions to identify ESC issues from the standpoint of the investigators and program staff. We held 11 group discussions with a total of 168 investigators and program officers (see Table S1). We asked them about gaps in knowledge with regard to the key ESC issues affecting the ultimate adoption of the technologies arising from their projects. We used our knowledge of the proposals to ask follow-up questions about specific issues in the groups. One member of our team took detailed notes of these sessions, and these data were analyzed using content analysis to identify key ESC issues by their relevance to particular Grand Challenge goals (Figure 1). We presented the listing of ESC issues to the GCGH investigators and program officers at a member check, in a February 2006 activity report, in the June 2006 ESC newsletter, and in a volume of ESC working papers at the October 2006 annual meeting. The list was well received in all contexts.

Between May and September 2006, as part of a larger study on the key forces influencing the development and adoption of health biotechnologies in the developing world, we conducted semi-structured, face-to-face interviews with experts from the developing world in academia, government, civil society, and the private sector. A few of these interviews were conducted in small groups of two or three or over the telephone. Of the 99 interviewees we originally contacted based on input from GCGH project teams and staff, the ESC team, literature searches, and snowball sampling, 70 participated (see Table S2). While we received no response from a very small number of these invitees, most of those who were not enrolled were agreeable to being interviewed, but we had reached saturation in the data so did not proceed to interview them. Of the participants, 21% were female. Participants represented the following World Health Organization regions: Africa, the Americas, the Eastern Mediterranean, South-East Asia, and the Western Pacific. They came from government, academia, industry, and civil society. We asked participants to identify key ESC issues related to the GCGH technologies and then to comment on the ESC list generated by GCGH investigators and program officers.

Interviews maintained flexibility to pursue emergent issues, allowing informants to share information on the basis of personal insights and/or issues raised by other informants. Each key informant was provided in advance an overview of the GCGH program and a description of a number of relevant projects in order to promote informed discussion. The interviews took place in English, with Chinese interpreters in some instances. During the interview, participants were presented with the ESC issues identified by the GCGH principal investigators and program staff, as shown in Figure 1. They were asked if the list captured the ESC issues in the GCGH initiative, if there were any issues missing, any listed that should not be, or any that needed to be modified. Participants were also asked another set of questions about the factors (e.g., scientific, political, financial) that facilitate or impede development or adoption of health biotechnology in the developing world, and this analysis is reported elsewhere [10]. We audiotaped, transcribed, and verified the interviews, and then performed a content analysis of the data. All named informants gave permission to be quoted. Anonymous participants chose not to be linked to their quotations but agreed to be listed among the study participants.

Developing world experts confirmed the ESC issues identified by GCGH investigators and program officers. However, they also provided important modifications to the list shown in Figure 1:

1. Almost all ESC issues were seen to cut across almost all GCGH goals, so the issues are no longer categorized by goal.
2. The issue “unintended promotion of unsafe sexual practices” was expanded to apply to other GCGH goals as “unintended consequences.”
3. Three other ESC issues were added: “gender,” “corruption,” and “accessibility.”
4. “Communication strategy” (i.e., the media strategy for the GCGH projects) was removed as a separate ESC issue since this was seen to be part of community and public engagement.
5. “North–South collaboration” became “collaboration” to reflect the importance of South–South collaboration.
6. The ESC issue in Figure 1 entitled “Socio-economic and cost–benefit analysis of technologies” became the broader theme of “affordability,” which also incorporates ethical notions of equity.

This compilation of views from investigators and developing world experts is the first description of ESC issues for the GCGH initiative. To our knowledge, it is also the first analysis of ESC issues related to a large-scale science program in the developing world. In this article we outline the ESC issues identified by these key informants.

## What Is Already Known

As discussed in the first paper of this series [1], the Human Genome Project set a standard for addressing ESC issues in large-scale science projects. Subsequent large-scale projects—such as the International HapMap Project [2] and National Nanotechnology Initiative [3]—contain ESC programs. Certain ESC issues surface across all three projects: community engagement, public engagement, benefit sharing, economic impacts of technologies, cultural issues, and accessibility.

However, the GCGH ESC program is the first large-scale science project focused exclusively on ESC issues related to the developing world [1]. The ESC issues identified here should be of interest to the science and development community, especially to those focused on global health. Moreover, we hope our approach, and specifically the way we went about identifying ESC issues, will be useful to future large-scale science projects focused on the developing world.

## Our Findings

The developing world experts identified thirteen issues, discussed below and illustrated with direct quotations.

### Community engagement

As described in greater detail in the third article in this series, community engagement is the process of working collaboratively with relevant partners who share a common goal and interests [4]. Many GCGH projects require extensive interactions with host communities, and both investigators and developing world informants stressed the need for appropriate community engagement.

**Figure 1:**
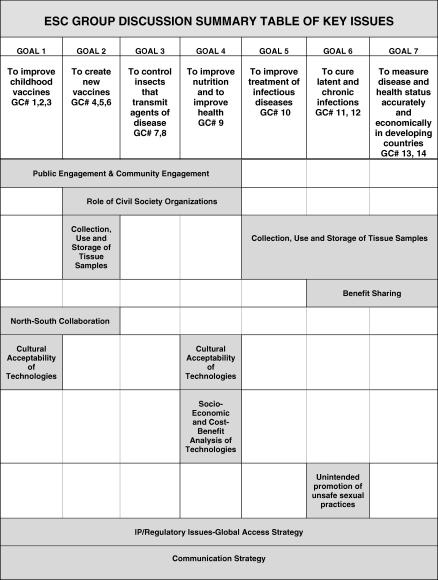
ESC Issues Identified during Group Discussions (Seattle, Washington, November 2005) GC, Grand Challenge; IP, intellectual property

For example, investigators introducing an intravaginal vaccine, conducting an observational study of severe cerebral malaria, or developing genetic strategies to control vectors will require adequate opportunities to listen to and understand the concerns and interests of the communities in which they work. Developing world experts stressed the need for early community engagement that involves the negotiations and deliberations necessary to make relationships between the investigators and community successful, respectful, and fair. “I think the most important lesson,” said Gita Ramjee, “is to engage the community right from the beginning, making sure that their issues are addressed, making sure that they have representatives at the highest level and at the lowest level, and just effective communication and ongoing transparency between the researchers and the community.” Community engagement seeks to prevent exploitation [5,6] and involves attention to community authorization, permission, ownership, and legitimacy. It also requires representativeness of community leadership, assent among community members, and the community's acceptance of new research strategies. As Dara Amar stated, “For sustainability of any program, it has to be owned by the community.”

### Public engagement

Public engagement is a process that provides people with trustworthy information on key policy issues, elicits their input, and integrates it into decision making and social action. Several of the GCGH projects could require investigators to assess public perceptions of and demand for new technologies. Developing world experts stressed that public engagement will be important in the introduction of nutritionally enhanced foods and genetic strategies to control mosquito populations. Based on experience with the introduction of genetically modified foods, GCGH investigators and developing world experts expect that some of the proposed studies will require well-developed strategies for engaging the public, gauging their views and concerns, and incorporating their insights into the implementation of the intervention [7]. “You have to somehow get the information through the radio, newspapers and the TV,” said Florence Wambugu. “But sometimes, to get to the grassroots you have to get to the community through schools and churches. We have to get to the people.” Respondents recognized that public engagement is a two-way process.

### Cultural acceptability

Some of the Grand Challenges involve the introduction of novel vaccine delivery systems, such as intravaginal or aerosolized vaccines, or the introduction of nutritionally enhanced foods that may differ (e.g., in color) from the varieties from which they are derived. In several cases, investigators identified possible cultural barriers to adopting these technologies. Developing world experts noted that researcher awareness of cultural issues stemming from gender, religion, historical context, sexual practices and contraception use, and the existence of a “culture of science” influence whether people who need a technology will use it. Calestous Juma argued that “Every technology is viewed first off in the context of how it might destroy the culture.” An anonymous participant linked this suspicion to colonialism: “Technology in Africa was introduced with colonization, and what the people of this continent were seeing is that colonial power will bring in only those technologies which were targeting natural resources…to sustain their own economies.” Gita Ramjee recommended: “Rather than shooting down the cultural beliefs, try to integrate the new technology in an appropriate way that doesn't impinge on their cultural beliefs.”

### Gender

Developing world experts identified gender as a critical ESC issue. They stressed the challenges of working with vulnerable populations such as commercial sex workers, girls, and adolescents, encouraging researchers to make efforts to empower their subjects against abuse and exploitation through education, skills training, and counseling. “We're researchers,” said Koleka Mlisana, who works with commercial sex workers in Durban, “but if you pick up an issue unrelated to your research topic like domestic violence what then is your obligation? Can we just turn our backs?”

The key informants noted that culturally engrained discrimination can leave women dependent on men, affecting their access to health care and contraceptives. Limited access to schooling for girls, in addition to a lack of discourse around the taboo topic of sex, prevents women from educating themselves about available reproductive health interventions. This discrimination, said informants, limits opportunities for women to fill gaps in the public health infrastructure, advocate for their needs, and make contributions in science and health. “I don't think we've had strong enough advocates for women's issues,” said Quarraisha Abdool Karim. “HIV... acutely highlights gender differences—greater burden of infection [in women], and also the North–South divide in that 90% of infections in women are really in developing countries and primarily in Sub-Saharan Africa.”

### Post-trial obligations/benefit sharing

Some GCGH investigators, particularly those proposing clinical trials or observational research with a clinical focus, identified issues related to benefit sharing with the host communities and individual research participants. Developing world experts stated that exploitative histories of trials in developing countries complicate investigator–subject relationships. “Very little of the good results of all the research that has been done in sub-Saharan Africa ever gets back to the people who took part in the studies,” said Godfrey Tangwa. Most participants in the GCGH group discussions and key informant interviews felt that investigators had a responsibility to ensure continued treatment for their subjects after a trial ends. “If you provide somebody with contraceptives, then you can not just take that away when the trial is over,” said Gita Ramjee. “One needs to look at sustainability of an intervention that is provided, and you cannot start something and take it away later.”

### Collaboration

Most of the GCGH projects involve collaborative research. Developing world experts emphasized the importance of capacity strengthening and collaboration between public and private sectors in the developing world, both to create sustainable science infrastructure and also to facilitate adoption of the resulting technologies. “International partnership is critical,” said M. K. Bhan, “not only to help find solutions but stimulate a culture in these emerging economies where, in future they could increasingly make a contribution to that [biotechnology innovation].” The key informants emphasized the importance of balanced power dynamics in North–South collaboration. Finally, they emphasized the importance of South–South collaboration in developing regionally relevant technologies and sustainable economic benefits. N. K. Ganguly commented that developing countries should be both the producers and the consumers of relevant health technologies: “These products need to move from the developing countries if they are meant for developing countries.”

### Role of civil society organizations

Both investigators and developing world informants highlighted the role of civil society and nongovernmental organizations (CSOs and NGOs) in working with communities, especially in developing countries, in the conduct of research and the uptake of technologies resulting from research findings. The fourth paper in this series explores further the role of CSOs in research [8].

In particular, investigators recognized that some of the GCGH projects might attract the attention of CSOs, and they were eager to identify constructive partnerships. “My experience with sleeping sickness, malaria and others has been that they [NGOs and CSOs] have been crucially important,” said Niresh Bhagwandin. Developing world experts noted that CSOs can play a critical role in improving delivery of health interventions with already established networks and rural outreach efforts. However, according to one anonymous participant, partnerships with NGOs are only successful when the missions of the collaborating organizations are aligned: “For example, some of the religious groups, if you talk to them about HIV, they put such a negative view…that they do more harm than good.” Other limitations arise when the NGO is not an accurate representative of community needs. Mpoko Bokanga commented on: “The NGO community…interposing itself between the research and the farmers, pretending that they know the needs of the farmers better than the researcher.”

### Affordability

Although GCGH investigators identified “socio-economic cost–benefit ratios” as an ESC issue, developing world experts expanded this theme into the broader notion of affordability. These experts saw affordability as an ethical issue rooted in the notion of equity. In populations living on less then a dollar a day, said participants, new technologies will not reach those in need unless they are donated or made affordable through subsidies.

“The cost really has to be affordable,” said Nares Damrongchai. “What if we were successful and really could deliver the solution in terms of technology solution, but the cost is too high and the people who really need the technology could not afford it…what kind of mechanism should we put in place to make the technology or the drug or the vaccine cheap enough to be affordable to people?”

### Accessibility

Ensuring accessibility, said developing world experts, requires adequate equipment and facilities staffed by competent health care workers. Many cited poor infrastructure, especially in rural areas, as leading to power cuts that break cold chains, inadequate roads and transport that prevent patients from reaching clinics, or a lack of potable water to administer pills. “In terms of adopting technology,” said Rosemary Wolson, “particularly in countries with large rural populations, the question of access to healthcare altogether is one that's very tricky.” Wolson described challenges such as “transport for people to get to hospital…refrigeration and cold chains…power cuts and roads.”

### Regulatory issues

Both investigators and developing world experts stated that weak or absent intellectual property frameworks discourage researchers from taking the risks that lead to innovation. “If we want to join the kind of the development ladder we have to agree that the intellectual property is something that we have to respect,” said Yongyuth Yuthavong. Regulatory structures must be balanced between ensuring consumer safety and encouraging product development and distribution. The absence of functioning regulatory regimes in the developing world diminishes both consumer trust and regional collaboration. As R. A. Mashelkar stated, “We [India] don't have a world class FDA [Food and Drug Administration]. We must build one progressively.” However, key informants said that for the world's most dire health emergencies, the human right to access essential medicines must supersede concerns about intellectual property. Yuthavong continued: “In certain cases, for example cases of drugs for AIDS, we have to stand firm that we cannot really allow the price to be the international price, we have to somehow make it cheaper, because there's a lot of people who are affected, and it's a question of humanity.”

### Collection, management, and storage of tissue samples

Many of the GCGH research projects require the collection, storage, and use of human tissue samples, and both investigators and developing world experts identified obstacles inherent in those processes. During the group discussion sessions, investigators recognized that guidance on the use of human tissues in research is still poorly developed, particularly for international collaborative research projects. Developing world experts noted that the lack of guidelines can hamper research, while overly strict regulations can have the same effect. You Lin Qiao said: “[The] only difficulty is the regulatory, the government…They have very, very strict rules, so any... biological material…you have to get it approved. Some experiments have to cancel or delay. It is not good for our research and scientific exchange.” Developing world experts also spoke to the need to develop guidelines that promote both scientific research and safety, and stressed that investigators must collaborate to enhance local research capacity to collect, manage, and store tissue samples [9]. An anonymous participant said: “If it [management, storage, and collection of tissue samples] can be done in South Africa and we have capacity to do it, we would do it…it's much better than having foreigners coming in.”

### Corruption and poor governance

Developing world experts cited corruption as an obstacle to accessing new technologies. In the media, Musthaque Chowdury identified a tendency to “play into the hands of some vested interests,” with media coverage “not necessarily based on facts.” The concentration of government power and lack of transparency in the approval and regulatory processes of new technologies persuaded some developing world experts that public health came second to political expedience. Informants with experience in civil society said that collaboration with the public sector breaks down when government partners demand bribes. They also stated that poor governance starves public health initiatives of funds and staff. Commenting on the 2006 Kenyan administration, Judi Wakhungu said: “That's the only time when we have a quorum in parliament, is when it is for their own benefit. So their salaries are absolutely exorbitant now, and then they've been in power since January 2003, and they have increased their remuneration three times.”

### Unintended consequences

GCGH investigators identified unintended promotion of unsafe sexual practices (e.g., increased exposure to sexually transmitted diseases due to perceived immunity as a result of GCGH vaccines or other technologies) as an ESC issue. Developing world experts saw broader application for this theme. Beyond the promotion of unsafe sex, participants identified unintended consequences stemming from gaps in knowledge regarding clinical trial site selection, phase III clinical trial outcomes, the effects of genetically modified organisms on biodiversity, local economy disruption, and drug resistance. Kiran Mazumdar-Shaw stated that unfamiliarity with the variables of a region when conducting clinical trials can “make a mess of the whole program.” An anonymous participant, discussing genetically modified foods, stated: “Of course, I want to be sure that people are safe in eating these crops and that they're not exposed to future disease problems. In curing one issue, we don't want to create a public health disaster…and we must take whatever safeguards we can to prevent cross-pollination and pollution of a gene pool.” As R. A. Mashelkar stated, “In science, there are unintended consequences,” and their ethical implications cannot be underestimated.

## Next Steps

We recognize that this study reflects only a small sample of highly educated developing world informants. However, as leaders and experts in their fields, their understanding of these issues brings experience and perspective that adds tremendous value to our research. Additionally, we recognize the limitations of group discussions as a methodology which may not permit all participants to cover all relevant issues comprehensively.

Notwithstanding the inherent limitations of the methodology and composition of group discussions and interviews, this study fills a gap in the literature as the first description of ESC issues in the GCGH initiative. This paper offers insights on thirteen ethical, social, and cultural issues, raising the unique perspectives of developing world experts. The findings may help guide other researchers in global health focused on the developing world, allowing them to identify and address key potential ethical concerns with greater clarity and efficiency. In the GSGH ESC program, we are now exploring many of the issues identified in greater depth through global case studies.

## Supporting Information

Table S1 Composition of Group Discussions (See Box 1 of the first paper in this series for details on the goals and grand challenges [1])
(75 KB DOC).

Table S2 Developing World Key Informants (111 KB DOC).

## Abbreviations

CSO: civil society organization
ESC: ethical, social, and cultural
GCGH: Grand Challenges in Global Health
NGO: nongovernmental organization
