# Supplementary material for: Grand Challenges in Global Health: Ethical, Social, and Cultural Issues Based on Key Informant Perspectives
Source: PLoS Med. 2007 Sep 11;4(9):e268. doi: 10.1371/journal.pmed.0040268 (PMC1989735; doi:10.1371/journal.pmed.0040268)
Supplement: Table S1 — (See Box 1 of the first paper in this series for details on the goals and grand challenges [1]) (75 KB DOC). [file pmed.0040268.st001.doc]

**Table S1.** Composition of group discussions (See Box 1 of the first paper in this series for details on the goals and grand challenges [1])

| **Discussion Group Number** | **Composition, by Grand Challenge** | **Number of Participants** |
| --- | --- | --- |
| 1 | **11** Create therapies that can cure latent infection  **12** Create immunological methods to cure latent infection | 15 |
| 2 | **9** Create a nutrient-rich staple plant species | 18 |
| 3 | **5** Design antigens for protective immunity | 14 |
| 4 | **6** Learn about immunological responses  **13** Develop technologies to assess population health  **14** Develop versatile diagnostic tools | 14 |
| 5 | **7** Develop a genetic strategy to control insects | 15 |
| 6 | **4** Devise testing systems for new vaccines  **12** Create immunological methods to cure latent infection | 15 |
| 7 | **1** Create effective single-dose vaccines  **2** Prepare vaccines that do not require refrigeration | 18 |
| 8 | **6** Learn about immunological responses  **10** Find drugs and delivery systems to limit drug resistance | 19 |
| 9 | **8** Develop a chemical strategy to control insects | 7 |
| 10 | **6** Learn about immunological responses | 13 |
| 11 | **3** Develop needle-free vaccine delivery systems | 17 |
