# Supplementary material for: Grand Challenges in Global Health: Ethical, Social, and Cultural Issues Based on Key Informant Perspectives
Source: PLoS Med. 2007 Sep 11;4(9):e268. doi: 10.1371/journal.pmed.0040268 (PMC1989735; doi:10.1371/journal.pmed.0040268)
Supplement: Table S2 — (111 KB DOC). [file pmed.0040268.st002.doc]

**Table S2. Developing World Key Informants**

| **NAME** | **TITLE & INSTITUTION** | **NAME** | **TITLE & INSTITUTION** |
| --- | --- | --- | --- |
| Mohamed Abdullah | Aga Khan University | N.K. Ganguly | Director General, Indian Council of Medical Research |
| Stephen Gaya Agong | Executive Director, African Academy of Sciences | Mohamed Hassan | Executive Director, Academy of Sciences for Developing World |
| Khalil Ahmed | Executive Director, Shantha Biotechnics Ltd. | Marian Jacobs | Dean, Faculty of Health Sciences, Univ. of Cape Town |
| Ademola Ajuwon | Senior Lecturer & Sub-Dean, Faculty of Public Health, University of Ibadan | Gerardo Jiménez-Sánchez | General Director  National Institute of Genomic Medicine |
| George Alleyne | Special Envoy of the Secretary General for HIV/AIDS in the Caribbean, United Nations | Calestous Juma | Director, Program on Science, Technology and Innovation, Harvard University |
| Dara Amar | Professor & Chair, Dept of Community Health, St. John's Medical College | Musimbi Kanyoro | Secretary General, World YWCA |
| Niresh Bhagwandin | Executive Manager: Strategic Research Initiatives, MRC | Quarraisha Abdool Karim | Assoc. Prof. of Public Health, University of KwaZulu-Natal |
| M.K. Bhan | Secretary, Department of Biotechnology (India) | Salim Abdool Karim | Director, CAPRISA |
| Mpoko Bokanga | Executive Director, African Agricultural Technology Foundation | Nathaniel Khaole | Professor, Faculty of Health Sciences, Univ. of Cape Town |
| Swami Brahmeshananda | Secretary, Ramakrishna Mission Ashrama, Chandigarh, India | Bruno Kilunga Kubata | Network Director, BioSciences Eastern and Central Africa |
| Zhijie Chang | Professor, School of Medicine, Tsinghua Univesity | Rajesh Kumar | Professor & Chair, Dept of Community Medicine, Post Graduate Institute for Medical Education and Research (India) |
| Mingzhe Chen | Professor of Medicine, Peking University | Ed Liu | Executive Director  Genome Institute of Singapore (GIS) |
| Mushtaque Chowdhury | Deputy Executive Director, BRAC | William Makgoba | Vice Chancellor, University of KwaZulu-Natal |
| John Clemens | Director General, International Vaccine Institute | R.A. Mashelkar | Director General, Council of Scientific and Industrial Research (India) |
| Nares Damrongchai | Executive Director, APEC Center for Technology Foresight | Kiran Mazumdar-Shaw | Chair and Managing Director, Biocon Ltd. |
| Krishna Ella | Chairman & Managing Director, Bharat Biotech Ltd. | John McDermott | Deputy Director General, Int’l Livestock Research Institute |
| Peter Folb | Chief Specialist Scientist of the Medical Research Council of South Africa | Koleka Mlisana | Project Director, CAPRISA |
| **NAME** | **TITLE & INSTITUTION** | **NAME** | **TITLE & INSTITUTION** |
| Farhat Moazam | Prof. & Chair, Centre of Biomedical Ethics and Culture, SIUT, Karachi, Pakistan | Olive Shisana | President & CEO, Human Sciences Research Council |
| Villoo Morawala-Patel | Founder & CEO, Avesthagen Technologies | Fang-Lin Sun | School of Medicine, Tsinghua University |
| Carlos Morel | Former TDR Director | Xuehai Tan | CEO and President, HD Biosciences Corp. Ltd. |
| John Mugabe | Advisor, New Partnership for Africa’s Development | Godfrey Tangwa | Associate Prof. of Philosophy, Univ of Yaounde, Cameroon |
| James Ochanda | Associate Professor in the Department of Biochemistry, University of Nairobi, and Coordinator Biotechnology Laboratory Unit. | Patrick Tippoo | Research & Development Manager, Biovac Institute |
| Gideon B.A. Okelo | Professor of Medicine, University of Nairobi | Cristina Torres | Coordinator, FERCAP-SIDCER |
| Onesmo ole-MoiYoi | Research Director, Int’l Centre of Insect Physiology & Ecology | V. K. Vinayak | President, Biopharmaceutical R&D, Panacea Biotech Ltd. |
| Father Sebastian Ousepparampil | Director, Catholic Health Association of India | David Wafula | Research Fellow, African Centre for Technology Studies (ACTS) |
| Vikram Patel | Wellcome Trust Senior Clinical Research Fellow, London School of Hygiene & Tropical Medicine & Sangath, India | Judi Wakhungu | Executive Director, African Center for Technology Studies |
| Adi Paterson | Group Executive, Dept. of Science & Technology | David Walwyn | Group Manager, Council for Scientific & Industrial Research |
| You-Lin Qiao | Chief & Professor, Dept of Cancer Epidemiology, Chinese Academy of Medical Sciences | Florence Wambugu | CEO, Africa Harvest Biotech Foundation International |
| Atta-ur Rahman | President Pakistan Academy of Sciences | Carolyn Williamson | Professor, Institute of Infectious Disease, Univ. of Cape Town |
| Asad Raja | Professor & Chair, Dept of Surgery, Aga Khan University | Rosemary Wolson | Intellectual Property Manager, Council for Scientific and Industrial Research (CSIR) South Africa |
| Rajkumar Ramesar | Professor & Head, Division of Human Genetics, University of Cape Town | Lan Xue | Professor & Associate Dean, School of Public Policy and Management, Tsinghua Univ. |
| Gita Ramjee | Director  HIV Research MRC | Huanming Yang | Professor and Director, Beijing Genomics Institute |
| Jaime Sepulveda | Coordinator of the National Institute of Health in Mexico | Wang Yu | Director, Chinese Center for Disease Control and Prevention |
| Ismail Serageldin | Former VP of World Bank; Director of Library of Alexandria | Yongyuth Yuthavong | President, Thai Academy of Science and Technology |
| Yiming Shao | Chinese AIDS Expert | Xiaomei Zhai | Executive Director, Research Centre for Bioethics, Peking Union Medical College |
